# Mechanism of action of a bactericidal lipopeptide which targets lipopolysaccharides of the plant pathogen *Xylella fastidiosa*

**DOI:** 10.3389/fpls.2026.1953704

**Published:** 2026-10-05

**Authors:** Pau Caravaca-Fuentes, Gerard Riesco-Llach, Beatriz Gascón, Lidia Feliu, Marta Planas, Emilio Montesinos

**Affiliations:** 1 Laboratory of Plant Pathology (CIDSAV), Institute of Agrifood Technology, University of Girona, Girona, Spain; 2 LIPPSO, Institute of Agrifood Technology, Department of Chemistry, University of Girona, Girona, Spain

**Keywords:** antimicrobial peptide, membranolytic, microscopy, Nicotiana benthamiana, NMR

## Abstract

The mechanism of action of BP473, a BP100-derived undecalipopeptide, was characterized through the evaluation of its bactericidal activity against different *Xylella fastidiosa* subspecies and strains, the study of the relationship between antibacterial activity and lipopolysaccharides (LPS) interaction, the analysis of its binding to LPS by NMR spectroscopy, and the assessment of its antibiofilm activity. The effect of BP473 on pathogen cell ultrastructure and on plant defense responses was also determined. In addition, its capacity to inhibit *X. fastidiosa* infections was further assessed in *Nicotiana benthamiana* using three *X. fastidiosa* subspecies. BP473 displayed potent bactericidal activity against *subsp. fastidiosa* and *multiplex*, whereas lower activity was observed against *subsp. pauca*. In contrast, its antibiofilm activity was limited. The bactericidal activity of BP473 was significantly reduced in the presence of free LPS, while that of BP178 remained unaffected, demonstrating the interaction between BP473 and LPS. NMR studies confirmed that all BP473 residues contribute to LPS binding, with the acetyl group and the aromatic protons of Phe^4^ showing the strongest interactions. Additional key binding epitopes included the *meta* aromatic protons of Tyr^10^ and the δ-CH_3_ and γ-CH_2_ protons of Leu^3^ and Ile^7^, respectively. Ultrastructural analyses by SEM in STEM mode revealed that both BP473 and the fluorescently labelled derivative BP473-K(CF) caused severe membrane damage in bacterial cells, supporting a membranolytic mode of action. BP473 elicited a weak plant defense response after a two-day treatment, mainly activating early signaling components without inducing a strong salicylic acid-dependent response. Importantly, BP473 reduced *X. fastidiosa* infection *in planta*, although its efficacy varied among bacterial subspecies. Overall, these results indicate that BP473 acts primarily through membrane disruption and LPS binding, while providing strain-dependent protection against *X. fastidiosa* infection.

## 1 Introduction

*Xylella fastidiosa* is a xylem-limited Gram-negative plant-pathogenic bacterium that infects a wide range of plant species and causes severe diseases in crops of major agricultural and economic importance, including grapevine, olive, almond and citrus (Gibin et al., 2023; Purcell, 2013; Trkulja et al., 2022). The pathogen is transmitted by xylem sap-feeding insects and colonizes the xylem vessels, where bacterial proliferation and biofilm formation restrict water transport. This results in symptoms such as leaf scorch, branch dieback and, in severe cases, plant death (Giménez-Romero et al., 2023; Sicard et al., 2018; Sun et al., 2013).

Since its first major outbreak in Europe, in southern Italy, *X. fastidiosa* has become a major plant-health threat in several countries of the European Union, particularly in Mediterranean areas, where susceptible crops and insect vectors favor its spread (EFSA, 2019a; Olmo et al., 2021; Trkulja et al., 2022). Current management strategies rely mainly on quarantine, eradication or containment measures, restrictions on plant movement, and vector control. However, these approaches have limited or only transient efficacy once the pathogen is established or infects a plant host (Burbank, 2022; EFSA, 2019b; European Commission, 2020).

Functional peptides have emerged as promising compounds for the control of plant pathogenic bacteria and fungi (Badosa et al., 2022; Montesinos, 2023). These peptides generally display direct antimicrobial activity, usually through interaction with bacterial membranes. In addition, they may also interfere with specific pathogenicity-related processes or activate host plant defense responses (Montesinos, 2023). In recent years, several antimicrobial peptides with activity against *X. fastidiosa* have been identified. In particular, among them it can be highlighted: BP178, a multifunctional peptide with bactericidal activity and the ability to induce plant defenses; 1036, with both bactericidal and antibiofilm activity; BP100, which interferes with twitching motility; and FV7, with the capacity to activate defense responses in host plants (Baró et al., 2020a; Moll et al., 2021, 2022, 2024, 2025). These studies support the interest of peptide-based compounds not only as antimicrobial agents, but also as target-oriented compounds aimed at key biological processes involved in *X. fastidiosa* pathogenicity.

Among the bacterial targets that can be explored, lipopolysaccharides (LPS) are particularly relevant because they are major components of the outer membrane of Gram-negative bacteria and play key roles in membrane stability, host interaction, adhesion and virulence (Bertani and Ruiz, 2018; Newman et al., 2001). In *X. fastidiosa*, LPS contribute to immune evasion, cell-to-cell aggregation, and host colonization, making them major virulence factors and potential targets for the development of new agents to control this pathogen (Clifford et al., 2013; Rapicavoli et al., 2018b). Recently, our group identified BP100-derived lipopeptides with strong LPS-neutralizing capacity, high bactericidal activity against *X. fastidiosa*, and low toxicity (Caravaca-Fuentes et al., 2025). Among them, BP389, BP473, and BP475 combined high LPS interaction, strong bactericidal activity, and low toxicity. Remarkably, confocal microscopy revealed that the fluorescent derivative BP473-K(CF) interacted rapidly with *X. fastidiosa* cells. This behavior is consistent with a lytic effect and points out that BP473 is a promising candidate against *X. fastidiosa*, highlighting the importance of performing a more thorough study of its mode of action.

Based on the above, in the present work, we characterized the activity of BP473 against different *X. fastidiosa* subspecies and strains, evaluated the antibacterial activity-LPS interaction relationship, studied its LPS binding by NMR, and assessed its effect on biofilm formation. We further investigated the interaction of BP473 with bacterial cells and characterized the peptide-induced ultrastructural cell damage. Finally, we evaluated whether BP473 induces defense-related genes and controls *X. fastidiosa* infections in *Nicotiana benthamiana*. Globally, the aim of this work was to provide a comprehensive mechanistic basis for the activity of BP473 as candidate to control diseases caused by *X. fastidiosa*.

## 2 Materials and methods

### 2.1 Synthesis of peptides

Peptides were manually prepared by solid-phase synthesis using 2 or 5 mL polypropylene syringes fitted with a porous polyethylene disk. Solvents and reagents were removed by suction. The synthesis followed the standard 9-fluorenylmethoxycarbonyl (Fmoc)/*tert*-butyl (*t*Bu) strategy. A PAC-ChemMatrix resin (0.69 mmol/g) was used to prepare BP178 whereas a Fmoc-Rink-MBHA resin (0.56 mmol/g) was employed to synthesize 1036, BP100, BP473 and BP473-K(CF). Peptide chain assembly, consisting of sequential steps of Fmoc removal and amino acid coupling, as well as the incorporation of the butanoyl group, was performed as previously reported (Oliveras et al., 2021; Caravaca-Fuentes et al., 2021, 2025). BP473-K(CF) was prepared by incorporating 5(6)-carboxyfluorescein (CF) at the side chain of the N-terminus lysine residue, which was coupled as Fmoc-Lys(Mtt)-OH. Once the BP473 sequence was completed, the 4-methyltrityl (Mtt) group was removed with TFA/CH_2_Cl_2_ (1:99, 4 x 5 min) under stirring at room temperature. Next, 5(6)-carboxyfluorescein (2.5 equiv.) was preactivated with ethyl cyano(hydroxyimino)acetate (Oxyma) and *N,N*-diisopropylcarbodiimide (DIPCDI) in CH_2_Cl_2_/*N*-methyl-2-pyrrolidone (NMP) (1:9) for 10 min (Soler et al., 2015). The mixture was then added to the corresponding deprotected peptidyl resin and allowed to react overnight at room temperature protected from light by covering the syringe with aluminium foil due to the light sensitivity of 5(6)-carboxyfluorescein. Peptidyl resins were cleaved using a trifluoroacetic acid (TFA)/H_2_O/triisopropylsilane (TIS) mixture. After TFA removal and precipitation with diethyl ether, crude peptides were purified by reverse-phase column chromatography, freeze-dried, analyzed by HPLC, and characterized by mass spectrometry.

### 2.2 *Xylella fastidiosa* strains and growth conditions

The experiments were carried out in officially authorized laboratories operating under biosafety level II+ containment conditions, in accordance with the guidelines established by the European and Mediterranean Plant Protection Organization (EPPO, 2006) and the European Union (EFSA, 2018). *Xylella fastidiosa* strains are described in **Table 1**. The strains were stored in Pierce’s disease broth (PD2) (Davis et al., 1981) supplemented with 30% glycerol and maintained at -80 °C. When needed, the strain was cultured in buffered charcoal yeast extract (BCYE) agar plates (Wells et al., 1981) at 28 °C for 7 days. Colonies were scrapped from the plates and cells were cultured on fresh BCYE at 28 °C for 7 additional days. For liquid cultures used in antibiofilm assays, PD3 was used (Davis et al., 1981). Cell suspensions were prepared in sterile succinate-citrate phosphate (SCP) buffer for bactericidal experiments, and in sterile phosphate buffered saline (PBS) for LPS extraction and antibiofilm assays. Suspensions were adjusted to 10^8^ CFU/mL (Moll et al., 2021).

**Table 1:** *Xylella fastidiosa* strains used in this work.

*X. fastidiosa*	ST^1^	Strain^2^	Host and origin	Reference
subsp. *fastidiosa*	1	Temecula (ATCC 700964)	Vineyard, California (USA)	(Rodrigues et al., 2003)
	1	IVIA 5387.2	Almond, Mallorca (Spain)	(Moralejo et al., 2019)
	1	IVIA 5770	Vineyard, Mallorca (Spain)	(Arias-Giraldo et al., 2020)
subsp. *pauca*	53	CN-28	Olive, Apulia (Italy)	(Saponari, *not published*)
	53	DeDonno	Olive, Apulia (Italy)	(Saponari et al., 2017)
	80	XYL 1513-1	Almond, Ibiza (Spain)	*Not published*
subsp. *multiplex*	6	IVIA 5901.2	Almond, Alicante (Spain)	(Giampetruzzi et al., 2019)
	41	CFBP 8173	*Prunus*, Georgia (USA)	(Giampetruzzi et al., 2019)
	7	M12	Almond, California (USA)	*Provided by* Leonardo de la Fuente

1 ST, Sequence type.

2 ATCC, American Type Culture Collection; IVIA, Instituto Valenciano de Investigaciones Agrarias; CFBP, Collection Française de Bactéries Associées aux Plantes.

### 2.3 Plant material

For experiments involving plant material, *Nicotiana benthamiana* was used as the host plant due to its rapid growth, ease of handling, and susceptibility to several *X. fastidiosa* strains (Baró et al., 2022). Seeds were sown and plants were grown in a controlled growth chamber at 25 ± 2 °C under a 16 h light/8 h dark photoperiod and 60% relative humidity. After 15 days, seedlings were transplanted into 0.8 L pots containing a substrate mixture of BV35:kokofina:perlite (Burés S.A.U., Barcelona, Spain) and fertilized with Osmocote^®^ KB (2 g/L; Evergreen Garden Care, Fréjus, France). Plants were maintained under a 16 h light photoperiod at 25 ± 2 °C and 8 h dark at 20 ± 2 °C, with a minimum relative humidity of 60%. Plants were watered as needed and fertilized weekly with a 100 ppm NPK solution. Experiments were performed using plants aged 4–6 weeks. Throughout the experimental period, standard insecticide and acaricide treatments were applied to prevent the presence of vectors and pests.

### 2.4 Bactericidal activity

Bactericidal activity of the peptides BP473, BP100 and BP178 was determined using a contact test combined with viability quantitative PCR (v-qPCR), as previously described (Baró et al., 2020a). This method enables selective quantification of viable cells by excluding dead or membrane-compromised cells using propidium monoazide (PMA), a DNA-binding dye. The sensitivity, amplification efficiency, and standard curves of the v-qPCR assay for the different bacterial strains were previously established and are shown in **Supplementary Table 1**.

Peptides were solubilized in sterile Milli-Q water to a stock concentration of 1 mM and filter-sterilized through a 0.22 μM pore-size filter. Briefly, the bactericidal activity of the selected peptides was determined by a 3 h contact test at 25 °C using a suspension of *X. fastidiosa* adjusted to 10^7^ CFU mL^-^¹. PMA was added, and samples were incubated and processed following previously described protocols (Baró et al., 2020a). The reduction in cell viability was obtained as previously described (Moll et al., 2021). Peptides were tested at final concentrations of 25 and 12.5 μM.

### 2.5 Antibiofilm activity

Antibiofilm activity was determined by assessing the effect of peptides on biofilm formation by *X. fastidiosa* subsp. *fastidiosa* IVIA 5387.2, as previously described (Moll et al., 2021). BP473, BP100, BP178 and 1036 were tested. Briefly, peptides were added to bacterial suspensions (OD_600_ = 0.1 in PD3 medium) to final concentration of 3.1 μM. Negative controls (growth medium and PBS) and untreated controls were included. Plates were incubated at 28 °C with shaking for 5 days. Following incubation, bacterial growth was assessed by measuring the OD_600_. For biofilm quantification, wells were washed, stained with crystal violet (0.1%), and the dye was subsequently solubilized. Absorbance was measured at 595 nm. Antibiofilm activity was expressed as the ratio (R) between treated and untreated samples, as described previously (Moll et al., 2021).

### 2.6 LPS interference on peptide bactericidal activity

Extraction of LPS from *X. fastidiosa* subsp. *fastidiosa* IVIA 5387.2 was performed using a LPS isolation kit (MAK339, Sigma-Aldrich, United States) following the manufacturer recommendations and as previously described (Caravaca-Fuentes et al., 2025).

The culturability of *X. fastidiosa* was determined after a contact assay with peptide–LPS mixtures to determine whether LPS interaction affects peptide antimicrobial activity. Peptides (BP473 and BP178) were pre-incubated with LPS at different concentrations and then added to bacterial suspensions (≈10^7^ CFU/mL). After 1 h at 25 °C, samples were serially diluted and plated on solid PD2 medium (Davis et al., 1981). Plates were incubated at 28 °C for 5–10 days, and colonies were counted. Untreated controls at 0 and 1 h were included. Peptides were tested at a final concentration of 3.1 μM, and LPS concentrations ranged from 0 to 1000 EU/mL. Antimicrobial activity was expressed as the reduction in bacterial culturability relative to the 1 h untreated control and was calculated as the difference in log (CFU/mL).

### 2.7 NMR experiments in presence of LPS

The tr-NOESY experiment was performed at the Research Technical Services (STR) facilities of the University of Girona (UdG) with an Ultrashield Avance III 400 (9.4T) spectrometer. This experiment (mixing time = 400 ms) was acquired with 1 mM BP473 in H_2_O/D_2_O (9:1, pH = 3.0) to which 10 μM LPS were added. Commercial LPS from *E. coli* 0111:B4 (Sigma-Aldrich) were employed due to their purity and suitability for NMR studies, and because previous experiments showed comparable peptide-LPS neutralization profiles using *E. coli* and *X. fastidiosa* LPS (Caravaca-Fuentes et al., 2025). Proton-decoupled ^31^P-NMR experiments were also performed using an Ultrashield Avance III 400 (9.4T) spectrometer. The ^31^P-NMR LPS spectra (3072 scans with a spectral width of 40 ppm) were recorded using 500 μM in H_2_O/D_2_O (9:1) to which increasing amounts of BP473 (from a 5 mM stock solution) were added. Final peptide concentrations were 125, 250, 500 and 750 μM.

The characterization of BP473 in LPS and the saturation transfer difference (STD)-NMR experiments were performed at the Centres Científics i Tecnològics of the Universitat de Barcelona using an UltraShieldPlus Avance III 600 (14.1T) spectrometer from Bruker (^1^H, 600 MHz; ^13^C, 150 MHz) equipped with a cryoprobe. 750 μM BP473 and 7.5 μM LPS in D_2_O were used. The following experiments were recorded at 308 K: 1D ^1^H-NMR; 2D ^1^H-^1^H TOCSY (mixing time = 80 ms), 2D ^1^H-^1^H TOCSY (mixing time = 22 ms, equivalent to COSY) and 2D ^1^H-^13^C HSQC.

^1^H-STD-NMR (512 scans) experiments were acquired at 308 K with a saturation time of 2 s using 750 μM BP473 and 7.5 μM LPS in D_2_O. On-resonance and off-resonance frequencies were set at –4.0 and 40 ppm, respectively. The on-resonance spectrum was subtracted with the one recorded without LPS saturation (off-resonance) affording the STD spectrum in which only the signals of the peptide that received saturation from LPS were observed. The difference of intensity between the on-resonance (*I*_on-res_) and the off-resonance (*I*_off-res_) spectra was used to calculate the intensity caused by the saturation transfer (*I*_STD_= *I*_on-res_ - *I*_off-res_). The STD effect was quantified with the amplification factor (*A*_STD_), which was correlated with the proximity between a proton of the peptide and LPS using the formula: *A*_STD_ = [(*I*_off-res_ - *I*_on-res_)/*I*_off-res_] × peptide excess = (*I*_STD_/*I*_off-res_) × ([Peptide]/[LPS]).

### 2.8 Electron microscopy

The effect of BP473 and BP473-K(CF) on the cell ultrastructure of *X. fastidiosa* was determined by scanning electron microscopy (SEM) in STEM mode, as previously described (Baró et al., 2020a). Following a 3 h contact with peptides at 3.1 and 12.5 µM (including untreated controls), cells were collected by centrifugation and chemically fixed with glutaraldehyde in sodium cacodylate buffer. Samples were post-fixed, dehydrated through a graded ethanol series, embedded in epoxy resin, and ultrathin sections (60–80 nm) were obtained and stained with heavy metals. Samples were imaged using SEM-STEM, and images were analyzed to classify cells as viable (V) (no ultrastructural alterations), affected (A) (cytoplasmic retraction or intracellular condensation), or lysed (L) (cell envelope disruption or membrane debris). Total damage was defined as the sum of affected and lysed cells. The image analysis was performed by a single observer and was not blinded to treatment. Classification was performed across three independent microscopic fields per condition, with ≥200 cells evaluated per condition. Representative examples of each cell category are provided in **Supplementary Figure 1**, and the raw cell counts for each microscopic field and treatment are included in **Supplementary Table 2**. For each condition, the proportion of damaged cells and confidence intervals were calculated. Differences between conditions were assessed using a Chi-square test, and pairwise comparisons (treatments vs. control and between peptides at the same concentration) were performed using Fisher’s exact test. Observations were performed using a TESCAN CLARA SEM microscope (TESCAN Brno, Czech Republic) equipped with an integrated STEM detector, which enables high-resolution scanning transmission electron detection, at the STR-UdG facilities.

### 2.9 Plant defense elicitation

Gene expression of defense-related genes in *N. benthamiana* was analyzed following treatment with peptides BP473 and BP178, as previously described (Baró et al., 2022). Plants were treated by endotherapy, and leaf samples were collected 24 h post-treatment. Three biological replicates were obtained per treatment. Total RNA was extracted from frozen leaf tissue, treated with DNase, and reverse-transcribed into cDNA following standard protocols. Gene expression analysis was performed by quantitative PCR using SYBR Green. Eleven defense-related genes were selected, and actin was used as the reference gene (**Supplementary Table 3**). Reactions were conducted in duplicate under standard cycling conditions. Relative gene expression was calculated using the 2^-ΔΔCt^ method (Livak and Schmittgen, 2001), comparing treated samples to untreated controls and normalizing to actin expression. Results were expressed as fold change, considering values > 1.5 as upregulation and < 0.5 as downregulation. Statistical analysis was performed using REST software (Pfaffl, 2002).

### 2.10 Efficacy assays in *Nicotiana benthamiana*

The efficacy of peptide BP473 for controlling *X. fastidiosa* infections was determined in *N. benthamiana*, using BP178 as a reference (Baró et al., 2022). Two experiments were conducted: one with subsp. *fastidiosa* IVIA 5387.2, and a second one including four additional strains (*subsp. fastidiosa* IVIA 5387.2, *subsp. pauca* De Donno and XYL1513-1, and *subsp. multiplex* M12). For each treatment and strain, nine plants were used. A combined preventive (1 day before pathogen inoculation) and curative (3 days post-inoculation) endotherapy treatment was applied. Each plant received three peptide injections (10 μL each, 20 mM). Bacterial inoculation was performed by microinjection (three injections of 10 μL at 10^8^ CFU/mL). Plants were evaluated at 42 and 65 days post-inoculation (dpi) for symptoms severity and bacterial population levels. Disease severity was assessed using a standardized index based on symptom progression (Clifford et al., 2013). For bacterial quantification, plant samples were collected, and genomic DNA was extracted from selected tissues. Bacterial populations were determined by qPCR and expressed as log(CFU/g) by means of a calibration curve for each strain (Baró et al., 2022). When values of Ct>35 the level of *X. fastidiosa* was considered as the detection limit (3.55 log(CFU/g) for IVIA 5387.2; 2.45 log(CFU/g) for XYL1513-1; 3.46 log(CFU/g) for De Donno; and 2.65 log(CFU/g) for M12). The experimental design at 42 dpi was of 6 plants per treatment arranged in 3 replicates of two plants per replicate. At 65 dpi only 3 plants per treatment were used. Then, for the 42 dpi data, a one-way ANOVA followed by Duncan’s multiple comparison test was used in the first experiment, whereas simple-effects comparisons including strain, treatment, and their interaction were used in the second experiment. For the 65 dpi data, a non-parametric Kruskal-Wallis test was used in the first experiment, whereas Mann-Whitney tests were used for within-strain comparisons between treated and non-treated plants in the second experiment. Mean population levels, standard deviations, and confidence intervals were calculated for all plants within each treatment.

## 3 Results

### 3.1 Bactericidal activity of BP473 against *Xylella fastidiosa*

The bactericidal activity of BP473 was determined against seven representative *X. fastidiosa* strains, using peptides BP178 and BP100 as reference.

Overall, peptide activity varied depending on both concentration and bacterial subspecies (**Supplementary Figure 2**). At 25 μM, the three peptides showed similar activity patterns, particularly BP473 and BP178, generally exhibiting high bactericidal effects against *subsp. fastidiosa* and *multiplex*, while displaying lower activity against *subsp. pauca*. At 12.5 μM, differences between peptides were more pronounced and allowed better discrimination of their activity (**Figure 1**). At this concentration, BP473 showed higher bactericidal activity against most *subsp. fastidiosa* strains and against *subsp. multiplex* strain CFBP8173 than the control peptides. In contrast, BP473 was poorly active against *subsp. pauca*. Statistical analysis at 12.5 μM revealed significant effects of strain and peptide, as well as a significant strain × peptide interaction, indicating that peptide performance depended on the bacterial strain.

**Figure 1:**
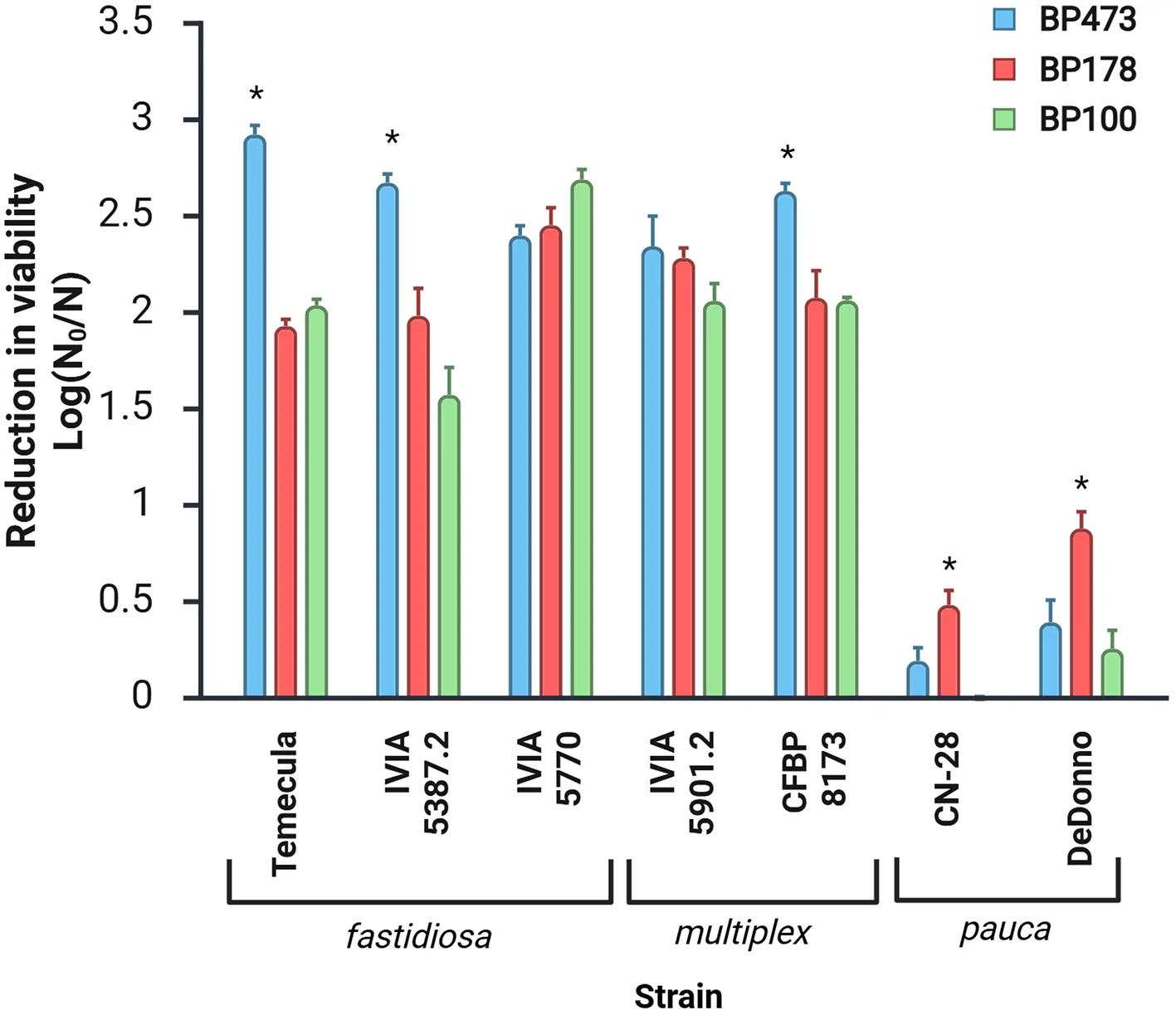
Bactericidal activity of BP473, BP178, and BP100 at 12.5 μM in seven strains of *X. fastidiosa.* The reduction in viability (logN_0_/N) based on v-qPCR is represented, where N_0_ is the pathogen concentration of the positive control and N the concentration after treatment. Bars are shown as the mean of three replicates and error bars represent the confidence interval (α = 0.05). A two-factor ANOVA (strain × peptide) was applied: significant strain effect (F = 1068.923, p < 0.0001), significant peptide effect (F = 119.447, p < 0.0001) and significant strain × peptide interaction (F = 41.846, p < 0.0001) where found. Given the interaction, Tukey’s test was performed within each strain; the asterisk indicates that the corresponding peptide bactericidal activity was significantly higher than the other two within the same strain (p < 0.05).

### 3.2 Antibiofilm activity of BP473

The antibiofilm activity of BP473 was compared to that of BP100 and BP178, as well as to that of peptide 1036, previously described to show high antibiofilm activity. Biofilm formation was quantified and expressed as the ratio between treatments and the non-treated control. BP100 and BP178 exhibited moderate antibiofilm effects (R (OD_600_(treatment)/OD_600_(NTC) = 0.39 and 0.34, respectively) and, as expected, peptide 1036 displayed the highest antibiofilm activity (R = 0.07). In contrast, BP473 showed a limited ability to reduce biofilm biomass, with values close to the non-treated control (R = 0.81).

### 3.3 Effect of LPS on the bactericidal activity of BP473

The effect of the presence of free LPS on the bactericidal activity of BP473 was evaluated. Peptide BP178, which lacks LPS-neutralizing capacity, was included in this assay for comparison purposes. These peptides were tested at 3.1 μM in the presence of increasing LPS concentrations (0–1000 EU/mL), and culturability was assessed after a contact test.

Two independent experiments were performed which showed consistent trends. **Figure 2** shows results of one of the experiments. BP473 exhibited a clear LPS concentration-dependent decrease in activity. In the absence of LPS, it reduced culturability by 1.5 log, whereas its effect was progressively attenuated at higher LPS concentrations, with a marked loss of activity at 1000 EU/mL. In contrast, BP178 maintained a high and stable bactericidal effect (2–3 log reduction) at all LPS concentrations. These results indicate that the presence of free LPS reduced the bactericidal activity of BP473, whereas that of BP178 remained unaffected.

**Figure 2:**
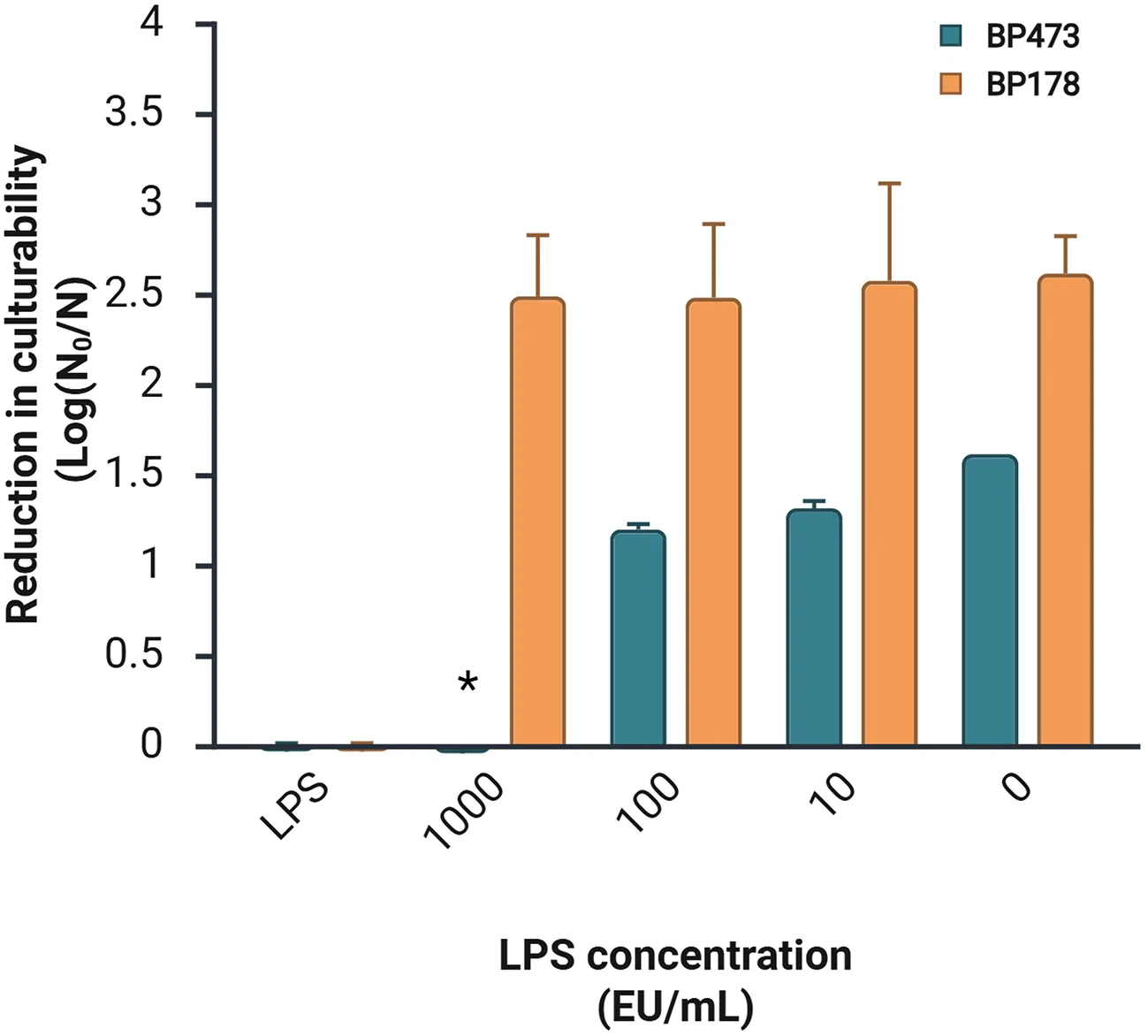
Reduction of culturability of *X. fastidiosa* subsp. *fastidiosa* IVIA 5387.2 after 1 h of contact with BP473 or BP178 (3.1 µM) in the presence of different LPS concentrations (0, 10, 100, or 1000 EU/mL). The LPS-only control (without peptide) showed no effect on bacterial viability. Bars represent the mean of three replicates. Error bars indicate the confidence interval (α = 0.05). Two-way ANOVA (Peptide × LPS) showed significant main effects of LPS (F = 74.5, p < 0.0001), peptide (F = 178.4, p < 0.0001), and their interaction (F = 19.7, p < 0.0001). Asterisks indicate significant differences compared to the other concentrations within the same peptide (Tukey, p < 0.05).

### 3.4 Evaluation of the binding of BP473 to LPS by NMR

BP473-LPS interaction was studied by transferred-NOESY (tr-NOESY). In the NOESY spectrum of BP473 recorded in aqueous medium no significant sequential NOEs were observed (**Figure 3A**), whereas the addition of 10 μM of LPS resulted in a substantial increase of tr-NOE connectivities. Not only all sequential αN(*i*, *i* + 1) tr-NOEs were detected, but also medium αN(*i*, *i* + 2), αN(*i*, *i* + 3), αN(*i*, *i* + 4) and even long-range correlations were found (**Figure 3B**). Intense tr-NOEs were observed between the acetyl group (~2.0 ppm) and the amide protons of Lys^1^, Lys^2^ and Phe^4^. In addition, aromatic protons of Phe^4^ and Tyr^10^ correlated with most of the side-chain protons of the amino acids, being especially intense those with methyl groups of aliphatic residues (Leu and Ile, **Figure 3C**). All this data confirms the interaction between BP473 and LPS and that, upon binding, BP473 undergoes a conformational restriction and stabilization in which hydrophobic interactions are important.

**Figure 3:**
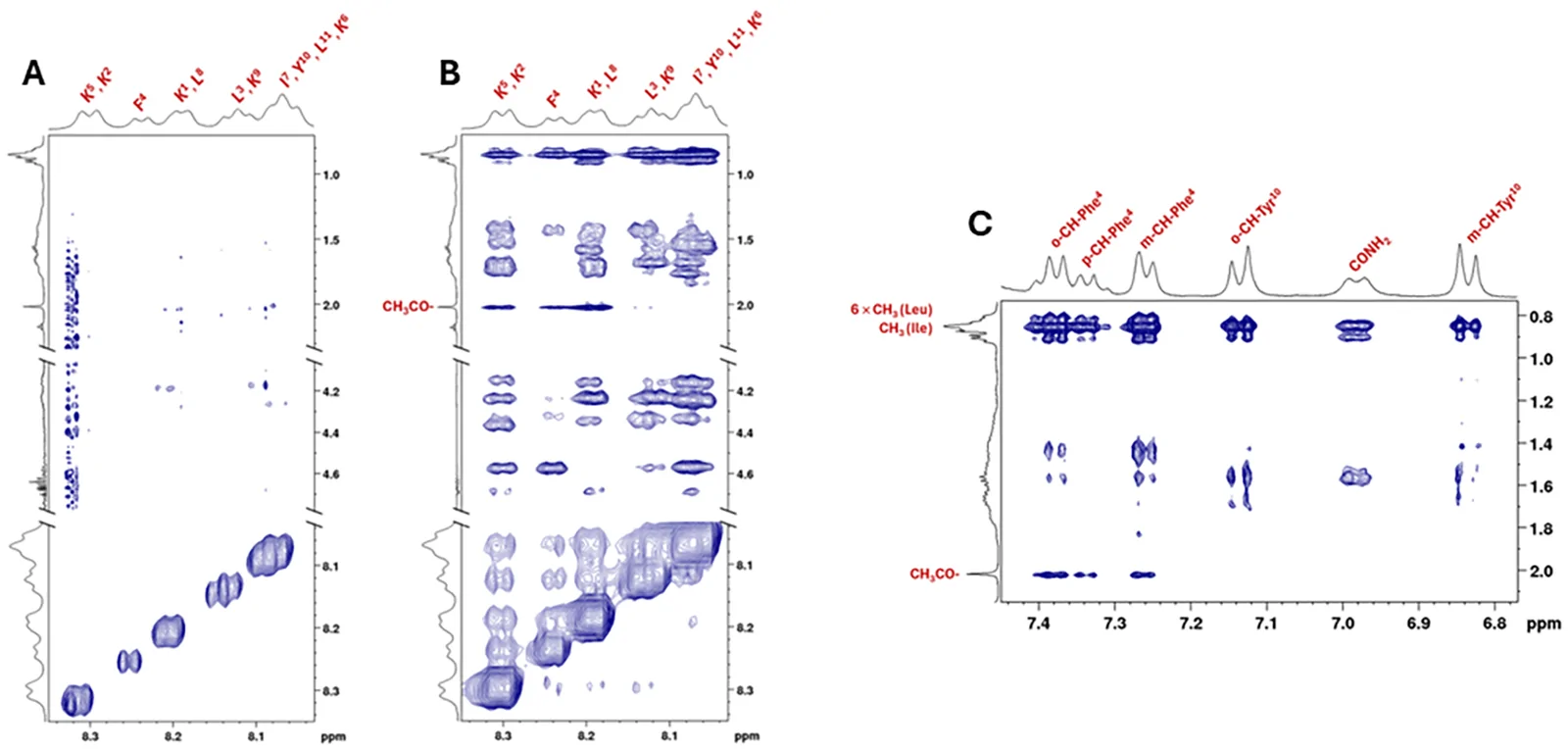
**(A)** NOESY of BP473 in aqueous solution in absence of LPS. **(B)** tr-NOESY of BP473 in presence of LPS. **(C)** tr-NOE signals of aromatic protons of BP473 in presence of LPS.

Furthermore, the effect of BP473 on LPS was studied by monitoring the changes in the ^31^P NMR spectrum of the signals corresponding to the phosphate groups of LPS upon addition of increasing amounts of BP473 (**Supplementary Figure 3**). As the molar ratio of BP473 increased, there were appreciable changes in the signal corresponding to the diphosphate groups at ~ –1.5 ppm. In particular, the ^31^P resonance of these groups slightly shifted downfield and underwent significant line broadening. These changes suggest that BP473 interacts with LPS and causes changes in the diphosphate groups. This interaction could probably occur through the cationic lysine residues of BP473.

To gain further insight into the interaction between BP473 and LPS and to identify the residues involved in this binding, STD-NMR experiments were conducted. It was observed that, after selective saturation of the LPS, the STD-NMR spectrum of BP473 (**Figure 4B**) was very similar to the reference spectrum (**Figure 4A**) demonstrating the significant interaction of BP473 with LPS. This experiment also revealed that multiple regions of BP473 are located in close proximity to LPS because no signals were missing.

**Figure 4:**
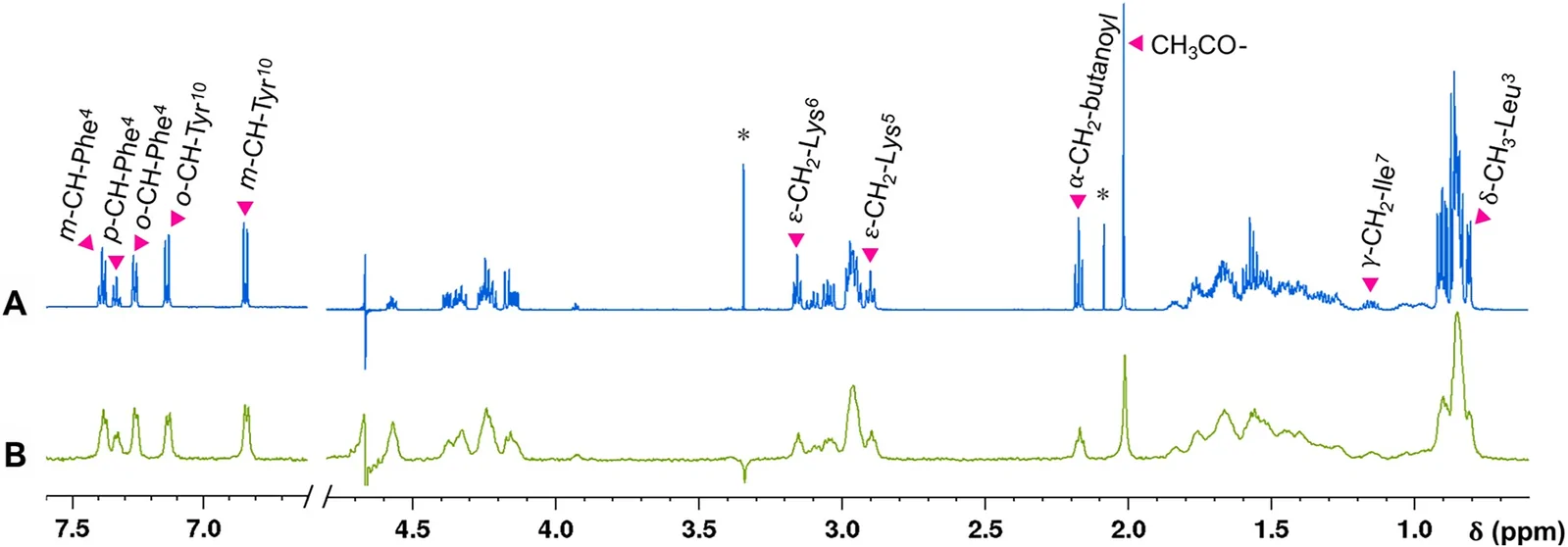
**(A)** Reference ^1^H-NMR spectrum of BP473 in LPS. The selected protons for epitope mapping are marked; **(B)** STD-NMR spectrum at a saturation time of 2.0 s (on-resonance = –4.0 ppm, off-resonance = 40 ppm). Asterisks mark signal of non-interacting impurities.

Next, the different signal intensities of the individual protons were studied to determine the binding epitope of BP473 to LPS. The analysis of the signals that were uniquely assigned was performed using the STD amplification factor (*A*_STD_) of the individual protons obtained at different saturation times (**Figure 5A**). It was observed that *A*_STD_ increased with the saturation time until 3 s, when a steady state was reached. The STD effects were more important for the aromatic protons of Phe^4^ and the N-terminus acetyl group. In contrast, the ϵ-CH_2_ groups of Lys^5^ and Lys^10^ exhibited low *A*_STD_. From these curves it can also be concluded that a saturation time of 2 s would be optimal for the observation of the STD effects. These saturation curves were fitted to the exponential function $A_{\text{STD}}={\text{STD}}_{\text{max}}\times (1-e^{-k_{\text{sat}}\;\times \;t_{\text{sat}}})$, where STD_max_ is the maximal STD intensity and *k*_sat_ is the saturation transfer rate constant.

**Figure 5:**
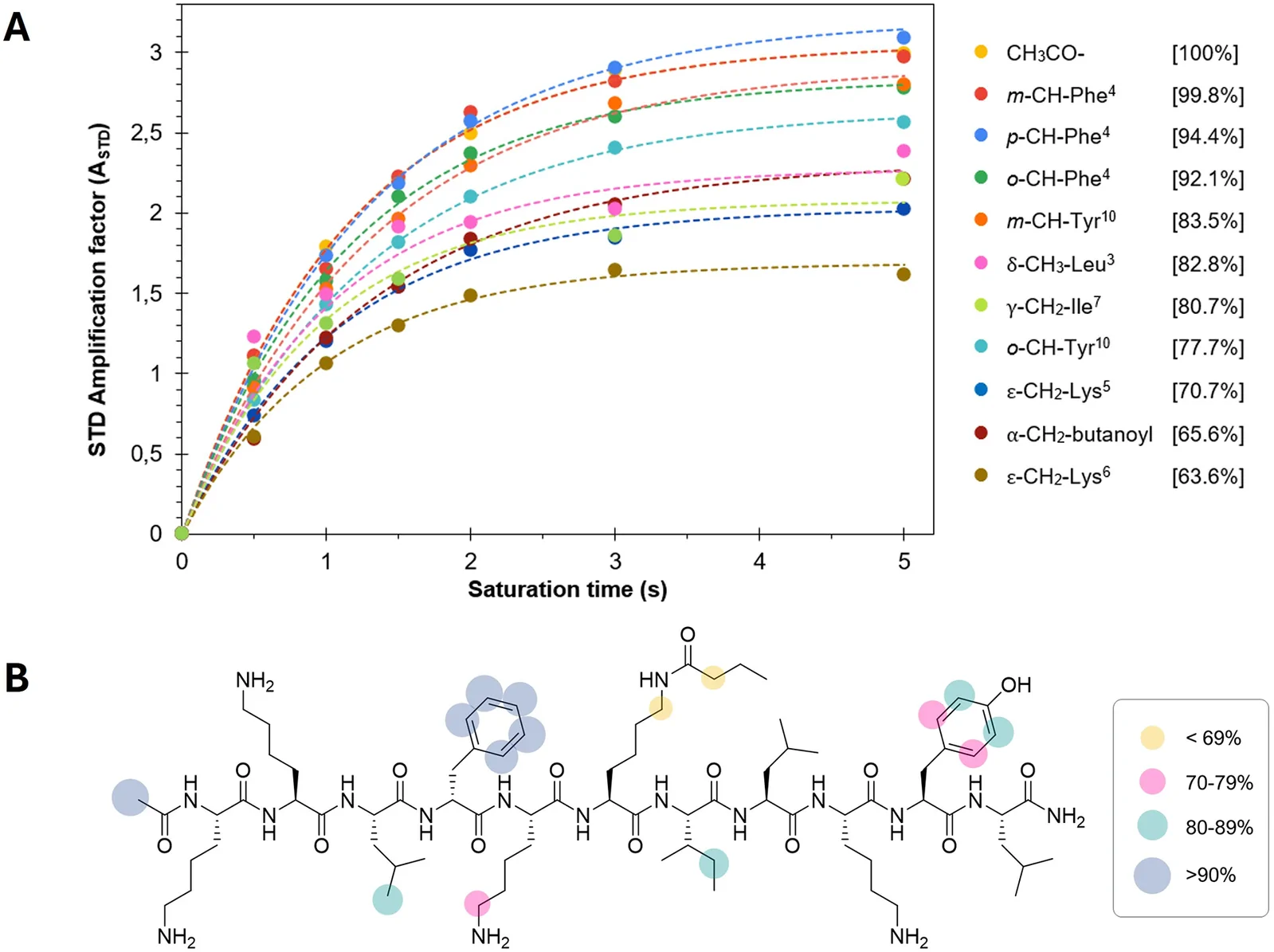
**(A)** A_STD_ values of selected protons of BP473 as a function of the saturation time. The relative degree of saturation of each proton normalized to that of the acetyl are included in brackets. **(B)** Epitope mapping obtained from the relative saturation of selected protons of BP473. The size of the circles reflects the proximity of the protons to LPS.

Next, the STD_total_ values for each proton were calculated (STD_total_ = STD_max_ × *k*_sat_). The highest STD_total_ corresponded to the CH_3_ of the N-terminus acetyl group and was assigned as 100%. The degree of saturation of all other protons was calculated with respect to t7his group. The STD_total_ values reflect more accurately the proximity of the peptide protons to LPS protons and allowed the identification of the binding epitopes of BP473 (**Figure 5B**). As previously observed, the acetyl group and the aromatic protons of Phe^4^ received the largest saturation transfer, with STD_total_ ≥ 92%. The aromatic protons at position meta of Tyr^10^ and protons δ-CH_3_ of Leu^3^ and γ-CH_2_ of Ile^7^ also showed a strong saturation transfer with STD_total_ values ranging from 81 to 84%. Protons of ϵ-CH_2_ received a moderate effect (STD_total_ = 70%) and the protons of Lys^6^ (ϵ-CH_2_ and α-CH_2_-butanoyl) exhibited lower STD_total_ values (63 and 65%, respectively).

### 3.5 Ultrastructural effects of BP473 and BP473-K(CF) on *X. fastidiosa*

The effects of BP473 and BP473-K(CF) on the ultrastructure of *X. fastidiosa* were analyzed by SEM in STEM mode. Qualitative observations revealed clear differences between untreated and peptide-treated *X. fastidiosa* cells (**Figure 6**). Untreated cells showed the typical rod-shaped morphology, with a well-defined and continuous cell envelope, homogeneous electron-dense cytoplasm, and no evident signs of structural damage. In contrast, cells treated with BP473 displayed progressive ultrastructural alterations mainly associated with the bacterial envelope. These included irregular or locally disrupted membranes, loss of cytoplasmic homogeneity, areas of cytoplasmic retraction, vesicle-like structures emerging from the cell surface, and extracellular material surrounding damaged cells. In some cells, the envelope appeared partially collapsed or discontinuous, suggesting advanced membrane destabilization. Treatment with BP473-K(CF) produced a similar pattern of damage, although alterations were frequently more evident. Cells exposed to this fluorescent derivative showed marked envelope disruption, extensive cytoplasmic leakage, accumulation of extracellular debris, and empty or ghost-like cells, consistent with cell lysis. Overall, both BP473 and BP473-K(CF) induced ultrastructural damage compatible with a membranolytic effect, and the presence of the CF label did not substantially modify the type of cellular alterations observed.

**Figure 6:**
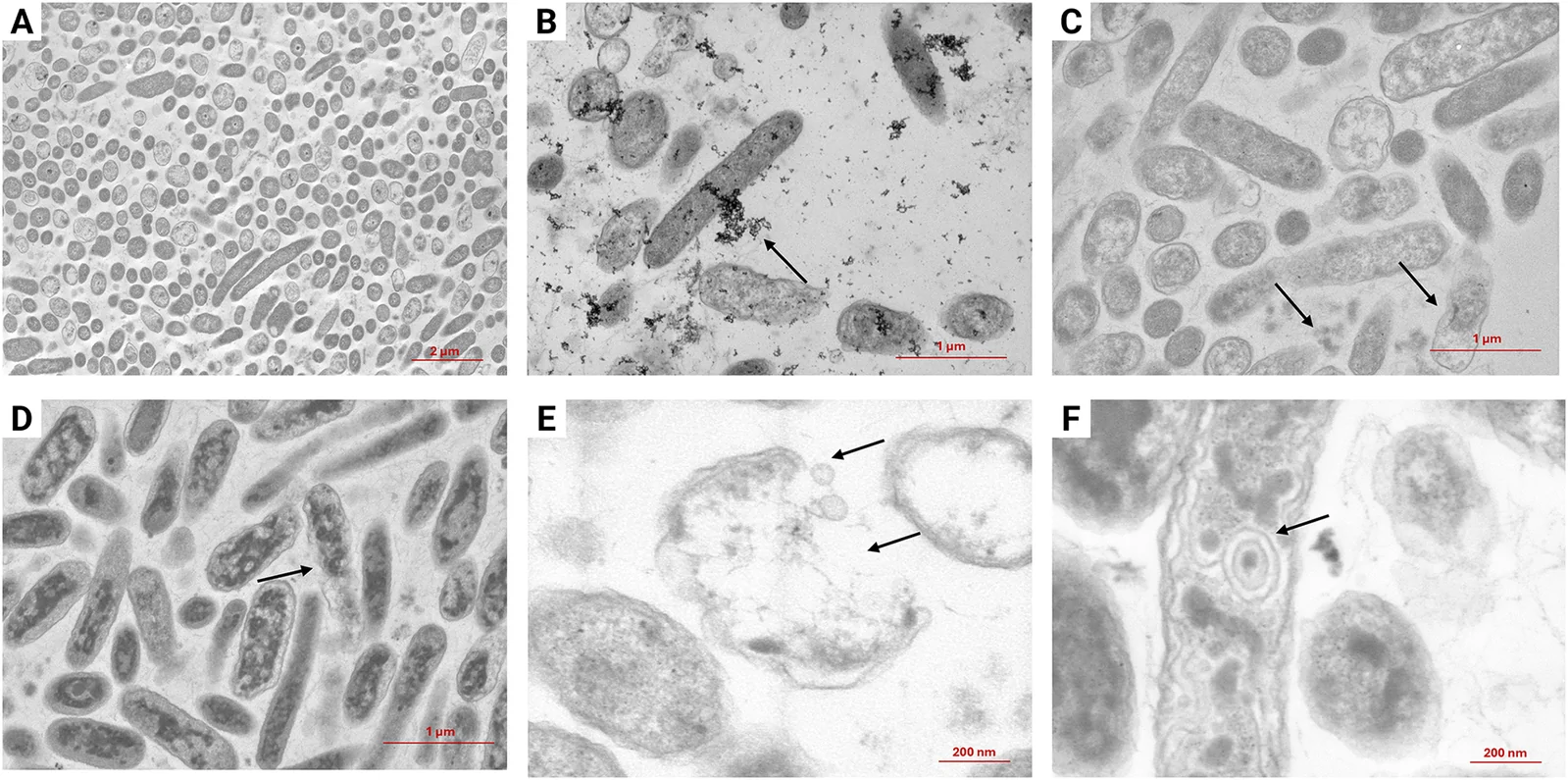
Images of *X. fastidiosa* subsp. *fastidiosa* IVIA 5387.2 (SEM in STEM mode). **(A)** corresponds to the non-treated control (NTC). **(B, C)** show cells after treatment with BP473, while **(D–F)** correspond to treatment with BP473-K(CF), all at 12.5 µM. Arrows indicate details of cellular damage.

Quantitative analysis of the electron microscopy images confirmed the qualitative observations and showed a concentration-dependent effect of both BP473 and BP473-K(CF) on *X. fastidiosa* cell integrity (**Figure 7**). Untreated samples were mainly composed of viable cells, which represented approximately 80% of the total population, whereas only a small proportion of cells showed altered or lysed morphologies. At 3.1 μM, both BP473 and BP473-K(CF) significantly increased the proportion of damaged cells compared with the untreated control. However, the overall effect at this concentration was moderate, with total damaged cells, including affected and lysed cells, representing approximately 10-15% of the population, and no significant differences were observed between the two peptides. At 12.5 μM, the proportion of damaged cells increased markedly. BP473 caused extensive cellular damage, with more than 90% of cells classified as affected or lysed, indicating a strong loss of cell integrity. In the case of BP473-K(CF), treatment resulted in an almost complete disappearance of viable cells and a significantly higher proportion of lysed cells, indicating that most bacteria had undergone severe envelope disruption. Overall, these results demonstrate that BP473 and BP473-K(CF) induce dose-dependent damage in *X. fastidiosa* cells, progressing from moderate membrane alterations at low concentration to extensive cell lysis at higher concentration.

**Figure 7:**
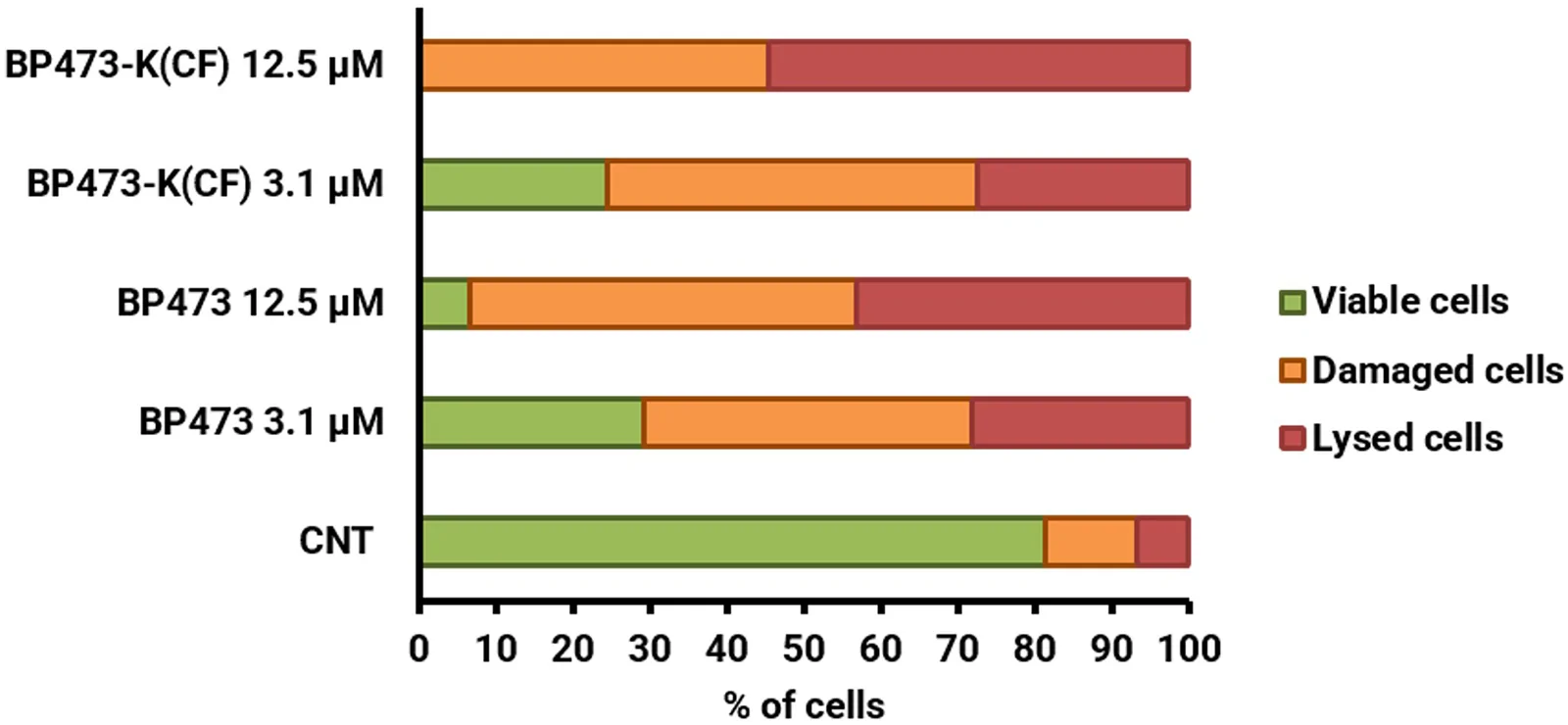
Distribution of viable (green), damaged (orange), and lysed (red) cells of *X. fastidiosa* IVIA 5387.2 observed by SEM in STEM mode after 3 h of contact with the indicated treatments. Bars represent the aggregated percentage composition per condition (≥200 cells per condition, 3 fields). A dose-dependent decrease in viable cells and an increase in damaged/lysed cells were observed; at BP473-K(CF) 12.5 µM, no viable cells were detected and lysis predominated. Overall differences between conditions were significant (χ² = 1548.8; p < 0.0001).

### 3.6 Effect of BP473 on host defense gene expression

The ability of BP473 to induce plant defense responses was determined in *N. benthamiana* by analyzing the expression of defense-related genes at 24 h post-treatment. BP178 was included as a reference peptide with known defense-inducing activity. Gene expression was quantified as fold change relative to untreated controls.

BP178 elicited a wide and strong response, with marked upregulation of *PR1*, *PR2*, and *PR3*, as well as induction of *LOX* and *MAPK3*, consistent with activation of both salicylic acid (SA) and jasmonic acid (JA)/ethylene (ET) pathways (**Table 2**). In comparison, BP473 induced a weak and selective transcriptional response. Significant upregulation was observed for *9LOX* and *MAPK3*, indicating activation of early signaling pathways and oxylipin-related responses. *PR4* and *PR4a* were significantly downregulated, while other defense-related genes, including *PR1*, *PR2*, and *PR3*, remained largely unchanged. Overall, these results indicate that BP473 does not trigger a strong activation of SA-dependent-dependent pathways and acts as a weak inducer of plant defenses, primarily activating early signaling components.

**Table 2:** Effect of BP473 and BP178 on gene expression in *N. benthamiana* at 24 h.

Genes	NTC	BP178	BP473
*ERF1*	1.09	1.58***	1.48
*9LOX*	1.07	2.24***	2.93***
*PR3*	1.05	4.07***	0.74
*PAL*	1.01	1.40*	1.39*
*PAL2*	1.01	0.76*	0.90
*PR1*	1.08	23.78***	1.27
*PR2*	1.17	7.20***	1.36
*PR4a*	1.05	0.82	0.43**
*WRKY25*	1.03	1.05	1.31*
*MAPK3*	1.07	1.63***	2.08***
*PR4*	1.02	0.63*	0.43**

Values represent fold change (FC) (2^-ΔΔCt)^, normalized to actin and referenced to the control (NTC). An increase was defined as FC > 1.5 and a decrease as FC < 0.5; values between 0.5 and 1.5 were considered sub-threshold changes. Asterisks indicate significance according to REST 2009: *** = significant upregulation (p < 0.05), ** = significant downregulation (p < 0.05). * = p < 0.05 but sub-threshold changes. Cells without asterisks correspond to non-significant changes (p ≥ 0.05).

### 3.7 Efficacy of BP473 in controlling *X. fastidiosa* infection in *N. benthamiana*

The efficacy of BP473 was evaluated in *N. benthamiana* using a combined preventive and curative treatment strategy. An initial experiment with *X. fastidiosa* subsp. *fastidiosa* IVIA 5387.2 was conducted to validate the approach and compare BP473 with the reference peptide BP178. Disease severity and bacterial population levels were assessed at 42 and 65 dpi (**Figure 8**). For symptom severity, significant differences among treatments were detected at 42 dpi (p = 0.002), but not at 65 dpi (p = 0.097). For bacterial population levels there were no significant differences among treatments at 42 dpi (p = 0.124), whereas significant differences were detected at 65 dpi (p = 0.037). These results indicated that there is some effect of the treatments but there were no consistency between severity and population levels across time.

**Figure 8:**
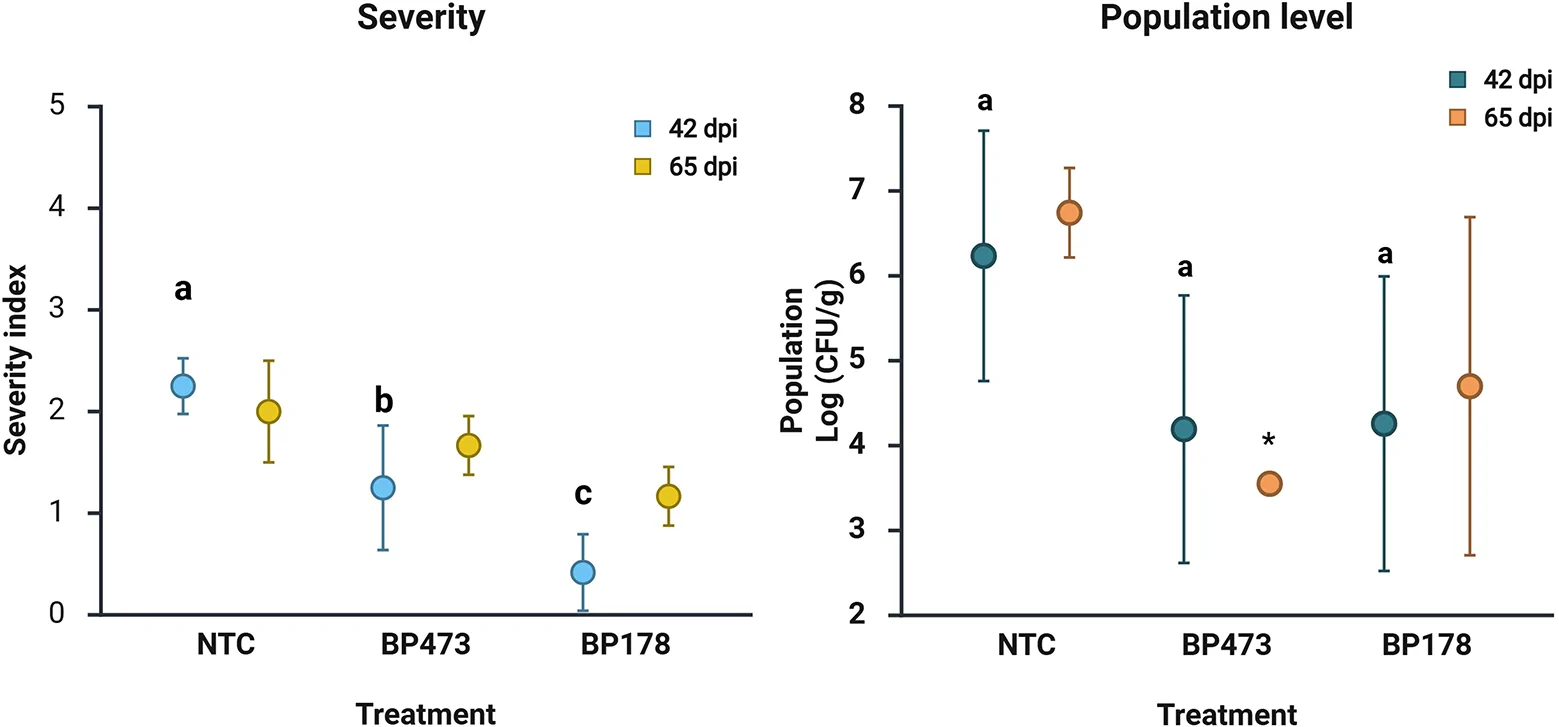
Effect of BP473 and BP178 on *X. fastidiosa* subsp. *fastidiosa* IVIA 5387.2 infection in *N. benthamiana*. Left panel: Symptom severity in untreated plants (NTC) or plants treated with BP473 or BP178 at 20 mM, evaluated at 42 and 65 dpi. Right panel: Bacterial population levels in zone A1 determined by qPCR and expressed as log(CFU/g). Points represent means and error bars indicate 95% confidence intervals (42 dpi: n = 6 plants; 65 dpi: n = 3 plants). At 42 dpi, different letters indicate significant differences among treatments according to one-way ANOVA followed by Duncan’s multiple comparisons test (p < 0.05). At 65 dpi, significant differences among treatments according to the Kruskal-Wallis test are indicated by an asterisk (*; p < 0.05).

A second experiment including multiple strains from different subspecies was performed. The efficacy of BP473 varied depending on the bacterial strain (**Figure 9**). Regarding severity, at 42 dpi, no overall significant effects were observed, although strain-specific trends were detected. At 65 dpi, within-strain comparisons showed that BP473 significantly reduced the symptom severity in plants inoculated with strains IVIA 5387.2 and DeDonno, whereas no significant differences between treated and non-treated plants were detected for the other strains. Bacterial population analysis at 65 dpi further supported the variability in the response to BP473. The peptide reduced bacterial populations to below the detection limit in plants inoculated with IVIA5387.2 and XYL1513-1, whereas its effects on M12 and DeDonno were variable or not significant. Overall, this data points out that the response to BP473 differed among *X. fastidiosa* strains evaluated.

**Figure 9:**
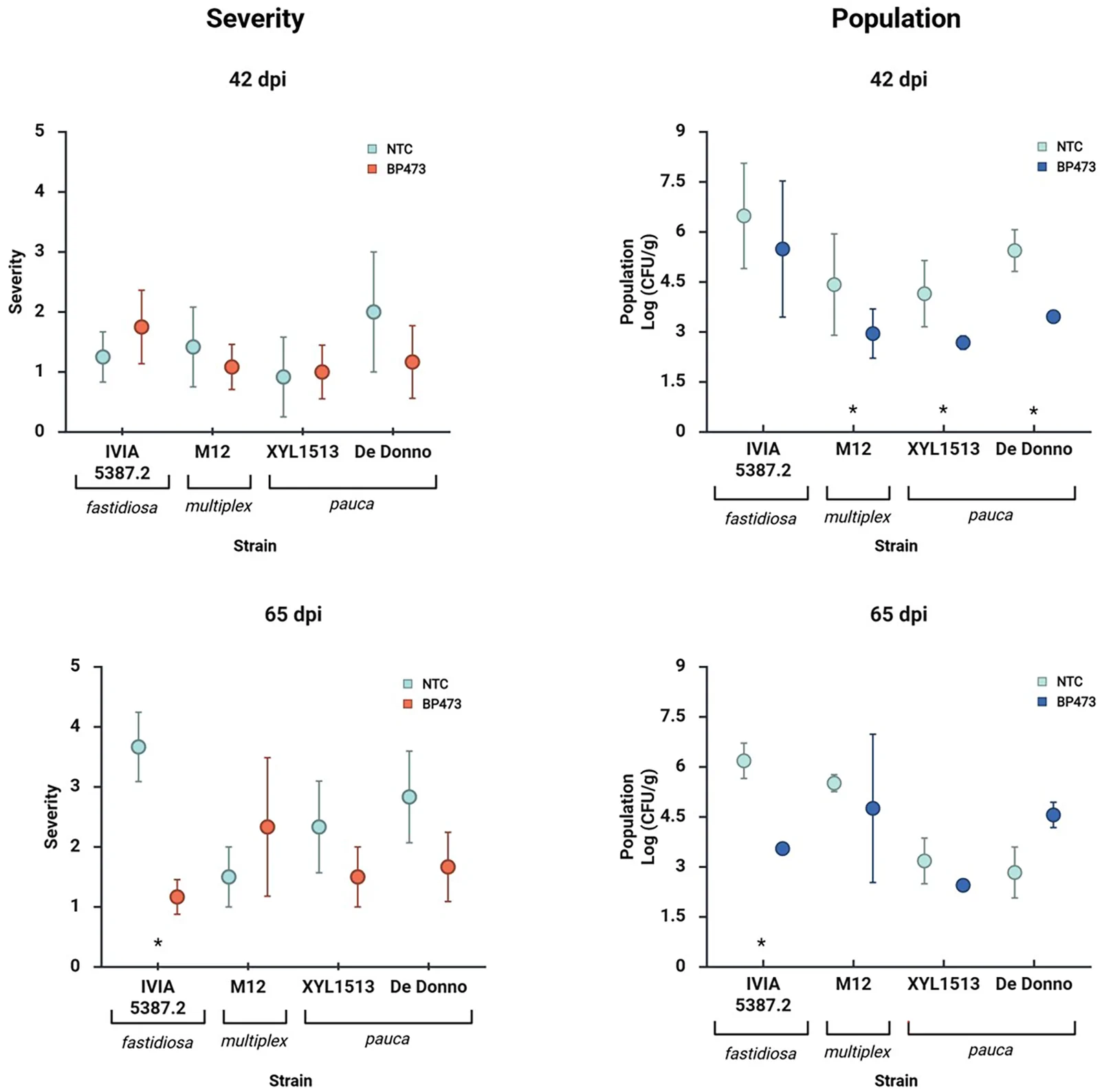
Effect of BP473 on infections caused by different *X. fastidiosa* strains in *N. benthamiana*. Left panels: Symptom severity and Right panels: Bacterial population levels in zone A1, determined by qPCR and expressed as log(CFU/g), in untreated plants (NTC) or plants treated with BP473 at 20 mM. Strains included IVIA 5387.2, M12, XYL1513-1, and De Donno. Points represent means and error bars indicate 95% confidence intervals (42 dpi: n = 6 plants; 65 dpi: n = 3 plants). At 42 dpi, asterisks (*) indicate significant differences between NTC and BP473 within each strain based on simple-effects comparisons (p < 0.05). At 65 dpi, asterisks (*) indicate significant differences between NTC and BP473 within each strain according to the Mann-Whitney test (p < 0.05).

Taking together, these results indicate that BP473 can limit *X. fastidiosa* infection *in planta*, although its efficacy is dependent on the bacterial strain. The combined analysis of symptom severity and bacterial population suggests a consistent antibacterial effect for some strains (e.g., IVIA 5387.2), while for others the response may involve additional or more complex interactions.

## 4 Discussion

Functional peptides represent a promising strategy for controlling *X. fastidiosa*, a xylem-limited pathogen for which effective treatments remain scarce. Among them, BP473 is a synthetic lipopeptide designed to combine strong bactericidal activity with capacity to interact with LPS, making it a relevant candidate for both mechanistic and applied studies (Caravaca-Fuentes et al., 2025).

The bactericidal activity of BP473 depended on the *X. fastidiosa* strain. Notably, this lipopeptide exhibited strong bactericidal activity against *X. fastidiosa* subsp. *fastidiosa* and subsp. *multiplex*, in some cases exceeding the bactericidal activity previously described for the bifunctional peptide BP178 (Baró et al., 2022). Conversely, the activity of BP473 against subsp. *pauca* was consistently lower. This differential susceptibility may be associated with strain-specific differences in cell envelope composition, including LPS structure and extracellular polysaccharides, which may hinder peptide access to the membrane in subsp. *pauca* (Baró et al., 2020b; Rapicavoli et al., 2018a; Scala et al., 2020). In fact, a previous study demonstrated that BP473 possesses high LPS neutralization capacity whereas BP178 does not bind to LPS (Caravaca-Fuentes et al., 2025). This difference in LPS interaction properties may contribute to the distinct bactericidal profiles observed, particularly the broad activity of BP178 against all *X. fastidiosa* subspecies compared with the more strain-dependent activity of BP473.

The interaction of BP473 with LPS was confirmed by interference assays using free LPS. Increasing LPS concentrations progressively reduced the bactericidal activity of BP473, whereas the activity of BP178 remained unaffected. These results indicate that BP473 interacts with LPS, which reduces its availability to exert its membranolytic activity. This behavior is consistent with NMR studies. In particular, tr-NOESY experiments, which are useful to analyze medium to weak binding interaction of short peptides with large biomolecules, showed a marked increase in NOE correlations upon addition of LPS, indicating conformational restriction and stabilization of BP473. In addition, the analysis of the STD effect of the selected protons suggests that the aromatic protons of Phe^4^ and Tyr^10^, and the aliphatic protons of Leu^3^ and Ile^7^ are the ones in closest proximity to LPS micelles. In contrast, the medium to low STD effects observed for the side-chain protons of Lys^5^ and Lys^6^ reflects the larger distance of these protons from LPS. However, ^31^P-NMR revealed that these cationic residues interact electrostatically with the negatively charged phosphate groups of LPS. These results can be rationalized considering that electrostatic interactions occur at larger distances than the hydrophobic forces (Park et al., 2009). These observations agree with those obtained with other lipopeptides, such as polymyxin B, in which the hydrophobic side chains are very proximal to the LPS during interaction (Bhunia and Bhattacharjya, 2011). Moreover, it was demonstrated that in this cyclic peptide the presence of cationic residues is essential for its binding to LPS and the presence of a long hydrophobic tail plays a crucial role in LPS neutralization facilitating its penetration into the lipidic outer membrane. In the case of BP473, the latter effect could not be observed because it bears an acyl group of only four carbons. A limitation of the present study is that the NMR analyses were conducted using *E. coli* LPS rather than LPS isolated from *X. fastidiosa*. Although *E. coli* LPS provided a robust model for structural studies, species-specific differences in LPS structure could affect peptide recognition and binding. Therefore, the NMR data obtained should be interpreted as describing BP473 interactions with a representative Gram-negative LPS model. However, the similar LPS-neutralization profiles previously obtained with *E. coli* and *X. fastidiosa* LPS (Caravaca-Fuentes et al., 2025) support that the NMR interactions observed with *E. coli* LPS may also occur in *X. fastidiosa*.

Microscopy analyses further supported the membranolytic mechanism. Confocal microscopy using the fluorescent analogue BP473-K(CF) demonstrated a quick peptide-cell association followed by signal loss consistent with lysis (Caravaca-Fuentes et al., 2025). SEM-STEM imaging revealed membrane disruption, cytoplasmic leakage, vesiculation and ghost cells. Quantitative analysis showed a dose-dependent transition from partially damaged cells to a near-complete lysis, consistent with a threshold-dependent membranolytic mechanism (Baró et al., 2020a; Sabri et al., 2024).

Despite its strong bactericidal activity, BP473 displayed low antibiofilm capacity. The lack of relationship between antibiofilm and bactericidal activities has been reported for other peptides, including BP178, and reflects that biofilm inhibition depends on mechanisms distinct from membrane disruption, such as interference with adhesion or quorum sensing (Batoni et al., 2016; De La Fuente-Núñez et al., 2016; Moll et al., 2021).

Regarding the capacity of BP473 of eliciting plant defense responses, a weak induction was observed after a two-day treatment. BP473 primarily activated early signaling components without triggering a strong SA-dependent response in *N. benthamiana*. In contrast, BP178 induced a broader activation of SA and JA/ET pathways, consistent with its role as defense elicitor (Baró et al., 2022; Montesinos et al., 2021). The response elicited by BP473 resembles that of certain natural lipopeptides that preferentially activate early signaling without full downstream defense activation (Ding et al., 2025; Farace et al., 2015).

*In planta*, BP473 controlled *X. fastidiosa* although efficacy was strain dependent. In the subsp. *fastidiosa* IVIA 5387.2, BP473 reduced bacterial populations to below detection levels at later stages, indicating sustained antimicrobial activity. There was a disconnection between bacterial load and disease severity as has been reported previously with other strains (Buisac et al., 2026). Its efficacy was weaker or more variable against other strains, including those belonging to subsp. *pauca*. Also, this strain-dependent response represents a relevant biological finding and highlights the need to consider strain diversity when evaluating peptide-based control strategies against *X. fastidiosa*. However, due to the limitations of the experimental design inherent to the reduced number of infected plants to perform the experiments under a biosafety greenhouse, it will be necessary to perform additional research to obtain more robust conclusions on the previous findings.

In addition, it should be noted that the assays were conducted in the model host *N. benthamiana*, which was selected because of its rapid growth, suitability for endotherapy treatments, efficient colonization by the pathogen, and the relatively short time required for symptom development (Baró et al., 2022; Buisac et al., 2026). Further studies in economically relevant natural hosts of *X. fastidiosa*, including olive, almond, grapevine, and citrus, as well as under greenhouse and field conditions, are therefore required before conclusions regarding the practical applicability of BP473 can be drawn. Nevertheless, previous studies have shown that related peptides, including BP178, KSLW-FV7 and BP100, are capable of reducing *X. fastidiosa* populations and/or disease severity in almond (*Prunus dulcis*) under greenhouse conditions (Moll et al., 2025).

Overall, this study indicates that BP473 acts through a membranolytic mechanism associated with LPS interaction. Based on our results, it can be postulated that electrostatic interactions between the cationic residues of BP473 and negatively charged phosphate groups of LPS, together with hydrophobic interactions involving aromatic and aliphatic residues, may contribute to peptide accumulation at the bacterial surface and subsequent membrane destabilization. Although the NMR data evidence the involvement of these interactions, the temporal sequence of the events remains to be established. Nevertheless, the dose-dependent ultrastructural damage observed, together with the loss of activity of the peptide in the presence of free LPS, is consistent with a mechanism in which LPS binding and membrane disruption play a central role in BP473 antibacterial activity. Thus, BP473 can be proposed to behave as an LPS-targeting lipopeptide with a membranolytic mechanism against *X. fastidiosa*.

## Data Availability

The datasets presented in this study can be found in online repositories. The names of the repository/repositories and accession number(s) can be found below: https://doi.org/10.34810/DATA3659.
