# Supplementary material for: Mechanism of action of a bactericidal lipopeptide which targets lipopolysaccharides of the plant pathogen Xylella fastidiosa
Source: Front Plant Sci. 2026 Oct 5;17:1953704. doi: 10.3389/fpls.2026.1953704 (PMC13682441; doi:10.3389/fpls.2026.1953704)
Supplement: Supplementary file 1 [file Table1.docx]

Supplementary Material

Supplementary Table 1. Standard curves for the calibration of v-qPCR assays with different *Xylella fastidiosa* strains.

| ***X. fastidiosa*** | **Strain^1^** | **Standard curve (combined)^2^** | **R2** | **Efficiency**  **(%)** | **Reference** |
| --- | --- | --- | --- | --- | --- |
| subsp. *fastidiosa* | Temecula | C_T_ = -3.64 log CFU/mL + 43.91 | 0.99 | 88.3 | (Moll et al., 2021) |
|  | IVIA 5387.2 | C_T_ = -3.56 log CFU/mL + 45.14 | 0.99 | 90.9 | (Moll et al., 2021) |
|  | IVIA 5770 | C_T_ = -3.81 log CFU/mL + 47.77 | 0.99 | 83.0 | (Moll et al., 2021) |
| subsp. *pauca* | CN-28 | C_T_ = -3.46 log CFU/mL + 44.23 | 0.98 | 94.6 | *This work* |
|  | DeDonno | C_T_ = -3.51 log CFU/mL + 43.82 | 0.99 | 93.0 | *This work* |
| subsp. *multiplex* | IVIA 5901.2 | C_T_ = -3.45 log CFU/mL + 44.60 | 0.99 | 94.9 | (Moll et al., 2021) |
|  | CFBP 8173 | C_T_ = -3.74 log CFU/mL + 45.34 | 0.99 | 85.0 | (Moll et al., 2021) |

^1^ IVIA, Instituto Valenciano de Investigaciones Agrarias; CFBP, Collection Française de Bactéries Associées aux Plantes
^2^Standard curves belong to a mixture of live cells at different concentrations (from 10 ^2^ to 10^8^ CFU/mL) and dead cells at 10^6^ CFU/mL.

Supplementary Table 2. Raw cell counts used for the quantitative STEM analysis.

| **Treatment** | **Concentration (µM)** | **Field** | **Viable (V)** | **Affected (A)** | **Lysed (L)** | | **Total cells** |
| --- | --- | --- | --- | --- | --- | --- | --- |
| **NTC** | - | 1 | 287 | 41 | | 20 | 348 |
| **NTC** | - | 2 | 260 | 32 | | 21 | 313 |
| **NTC** | - | 3 | 243 | 43 | | 24 | 310 |
| **BP473** | 3.1 | 1 | 73 | 80 | | 55 | 208 |
| **BP473** | 3.1 | 2 | 50 | 86 | | 66 | 202 |
| **BP473** | 3.1 | 3 | 57 | 96 | | 53 | 206 |
| **BP473** | 12.5 | 1 | 16 | 108 | | 80 | 204 |
| **BP473** | 12.5 | 2 | 20 | 99 | | 89 | 208 |
| **BP473** | 12.5 | 3 | 5 | 103 | | 97 | 205 |
| **BP473-K(CF)** | 3.1 | 1 | 55 | 92 | | 62 | 209 |
| **BP473-K(CF)** | 3.1 | 2 | 34 | 77 | | 35 | 146 |
| **BP473-K(CF)** | 3.1 | 3 | 69 | 143 | | 81 | 293 |
| **BP473-K(CF)** | 12.5 | 1 | 0 | 90 | | 113 | 203 |
| **BP473-K(CF)** | 12.5 | 2 | 0 | 93 | | 115 | 208 |
| **BP473-K(CF)** | 12.5 | 3 | 0 | 106 | | 121 | 227 |

Raw numbers of viable (V), affected (A), and lysed (L) *X. fastidiosa* subsp. *fastidiosa* IVIA 5387.2 cells counted in three independent microscopic fields for each treatment. Total corresponds to the total number of cells classified in each field. NTC: non-treated control

Supplementary Table 3. Genes and primers selected for gene expression analysis in *Nicotiana benthamiana*.

| **Gene** | **Gene code** | **Primer code** | **Sequence (5'-3')** | **Reference** |
| --- | --- | --- | --- | --- |
|  |  |  |  |  |
| **Lipoxygenase 9** | *9LOX* | Nb-9-LOX F  Nb-9-LOX R | ATATGTGCCAAGGGACGA  AATAGGCCTTCGCCATCA | (Huang & Schwab, 2011) |
|  |  |  |  |  |
| **Phenylalanine ammonia-lyase** | *PAL* | PAL F  PAL R | GTTATGCTCTTAGAACGTCGCCC  CCGTGTAATGCCTTGTTTCTTGA | (Wang et al., 2015) |
|  |  |  |  |  |
| **Ethylene responsive factor** | *ERF1* | ERF1 F  ERF1 R | GGCGAATTTTCCGGGAGACT  GGCTCCGATTTTACTTCGCC | (Abbas et al., 2018) |
|  |  |  |  |  |
| **PR-2, β 1,3-Glucanase** | *PR2* | PR2 F  PR2 R | GGGCTGTTAATTTGCAGTATCC  GGTTTATAACATCTTGGTCTGATGG | (Raffaello & Asiegbu, 2017) |
|  |  |  |  |  |
| **PR3, Endochitinase** | *PR3* | PR3 F  PR3 R | TGCCTTTTTCGGTCAAACTT  TGTAAATGGTTCTGCACTCAGG | (Raffaello & Asiegbu, 2017) |
|  |  |  |  |  |
| **PR-4, Barwin domain chitinase** | *PR4* | PR4a F  PR4a R | CAACCCACAGAACATTAACTGG  TTGTCGGCATCCCAAGTAGT | (Raffaello & Asiegbu, 2017) |
|  |  |  |  |  |
| **PR1, Pathogenesis related protein 1** | *PR1* | PR1 F  PR1 R | GGATGCCCATAACACAGCTC  GCTAGGTTTTCGCCGTATTG | (Obrępalska-Stęplowska et al., 2018) |
|  |  |  |  |  |
| **Phenylalanine ammonia lyase 2** | *PAL2* | PAL2 F  PAL2 R | GATTGGAGCTTTCGAAGACG  CGGTGATCGGACTCTTTCTC | (Yang et al., 2020) |
|  |  |  |  |  |
| **PR4, Pathogenesis related protein 4** | *PR4* | PR4 F  PR4 R | GGCCAAGATTCCTGTGGTAGAT  CACTGTTGTTTGAGTTCCTGTTCCT | (Villarroel et al., 2016) |
|  |  |  |  |  |
| **Mitogen-activated protein kinase 3** | *MAPK3* | MAPK3 F  MAPK3 R | GTCTGCTCGGTGTTGAATACGG  CCAATTACATTTTCATGGTCTAAATGGCG | (López et al., 2020) |
|  |  |  |  |  |
| **WRKY transcriptional factor 25** | *WRKY25* | WRKY25 F  WRKY25 R | CATGCGGTGGTCAGAAGCTG  CCGCTGGAACTAGTGGTGGT | (Ramos et al., 2021) |
|  |  |  |  |  |
| **Actin** | *Actin* | Actin F  Actin R | GTGAAGGAGAAGTTGGCTTAC  CTTCTGGGCAGCGGAATCTC | (Obrępalska-Stęplowska et al., 2018) |

Supplementary Figure 1. Representative SEM-STEM images illustrating the morphological criteria used for cell classification. **A:** Field containing predominantly morphologically intact cells (V), characterized by a bacillary morphology, continuous cell envelope, recognizable lamellar wall, homogeneous cytoplasm, and absence of membrane debris (NTC). **B:** Field containing predominantly affected (A) and lysed (L) cells, showing irregular cell contours, membrane blebbing/vesiculation, membrane debris, and ghost cells [**BP473-K(CF)**, 12.5 µM]. **C-D:** Representative affected cells (A), characterized by cytoplasmic retraction, enlargement of the periplasmic space, intracellular granularity/condensation, and membrane irregularities or blebs [**BP473-K(CF)**, 12.5 µM, and **BP473**, 3.1 µM, respectively]. **E-F:** Representative lysed cells (L), characterized by clear membrane discontinuity or collapse, extrusion of cellular contents, ghost cells, and adjacent free membranous material [**BP473-K(CF)**, 3.1 µM, and **BP473**, 12.5 µM, respectively]. Scale bars: 2 µm (A-B) and 200 nm (C-F).

Supplementary Figure 2. Bactericidal activity of **BP473**, **BP178**, and **BP100** against seven *X. fastidiosa* strains at 12.5 and 25 μM after a 3 h contact test. Viability reduction is expressed as log(N_0_/N), where N is the pathogen concentration after treatment and N_0_ is the concentration in the untreated control. Cells show the mean of three replicates per treatment; darker colors indicate higher reductions according to the color scale.

Supplementary Figure 3. ^31^P-NMR spectra of LPS at different molar ratios of **BP473**.

**References**

Abbas, H. M. K., Xiang, J., Ahmad, Z., Wang, L., & Dong, W. (2018). Enhanced Nicotiana benthamiana immune responses caused by heterologous plant genes from Pinellia ternata. *BMC Plant Biology*, *18*(1). https://doi.org/10.1186/s12870-018-1598-5

Huang, F. C., & Schwab, W. (2011). Cloning and characterization of a 9-lipoxygenase gene induced by pathogen attack from Nicotiana benthamiana for biotechnological application. *BMC Biotechnology*, *11*. <https://doi.org/10.1186/1472-6750-11-30>

López, M., Miranda, E., Ramos, C., García, H., & Neira-Carrillo, A. (2020). Activation of early defense signals in seedlings of nicotiana benthamiana treated with chitin nanoparticles. *Plants*, *9*(5). <https://doi.org/10.3390/plants9050607>

Moll, L., Badosa, E., Planas, M., Feliu, L., Montesinos, E., & Bonaterra, A. (2021). Antimicrobial Peptides With Antibiofilm Activity Against Xylella fastidiosa. *Frontiers in Microbiology*, *12*. https://doi.org/10.3389/fmicb.2021.753874

Obrępalska-Stęplowska, A., Zmienko, A., Wrzesińska, B., Goralski, M., Figlerowicz, M., Zyprych-Walczak, J., Siatkowski, I., & Pospieszny, H. (2018). The defense response of nicotiana benthamiana to peanut stunt virus infection in the presence of symptom exacerbating satellite RNA. *Viruses*, *10*(9). <https://doi.org/10.3390/v10090449>

Raffaello, T., & Asiegbu, F. O. (2017). Small secreted proteins from the necrotrophic conifer pathogen Heterobasidion annosum s.l. (HaSSPs) induce cell death in Nicotiana benthamiana. *Scientific Reports*, *7*(1). https://doi.org/10.1038/s41598-017-08010-0

Ramos, R. N., Martin, G. B., Pombo, M. A., & Rosli, H. G. (2021). WRKY22 and WRKY25 transcription factors are positive regulators of defense responses in Nicotiana benthamiana. *Plant Molecular Biology*, *105*(1–2), 65–82. https://doi.org/10.1007/s11103-020-01069-w

Villarroel, C. A., Jonckheere, W., Alba, J. M., Glas, J. J., Dermauw, W., Haring, M. A., Van Leeuwen, T., Schuurink, R. C., & Kant, M. R. (2016). Salivary proteins of spider mites suppress defenses in Nicotiana benthamiana and promote mite reproduction. *Plant Journal*, *86*(2), 119–131. https://doi.org/10.1111/tpj.13152

Wang, H., Yang, X., Guo, L., Zeng, H., & Qiu, D. (2015). PeBL1, a novel protein elicitor from Brevibacillus laterosporus strain A60, activates defense responses and systemic resistance in Nicotiana benthamiana. *Applied and Environmental Microbiology*, *81*(8), 2706–2716. https://doi.org/10.1128/AEM.03586-14

Yang, X., Lu, Y., Wang, F., Chen, Y., Tian, Y., Jiang, L., Peng, J., Zheng, H., Lin, L., Yan, C., Taliansky, M., MacFarlane, S., Wu, Y., Chen, J., & Yan, F. (2020). Involvement of the chloroplast gene ferredoxin 1 in multiple responses of Nicotiana benthamiana to Potato virus X infection. *Journal of Experimental Botany*, *71*(6), 2142–2156. https://doi.org/10.1093/jxb/erz565
